# Improving alcohol and substance use screening in school-age children: translation, adaptation and psychometric evaluation of the CRAFFT tool for Lumasaaba, Uganda

**DOI:** 10.1186/s13722-024-00465-7

**Published:** 2024-05-14

**Authors:** Joyce Sserunjogi Nalugya, Ingunn Marie Stadskleiv Engebretsen, Noeline Nakasujja, Grace Ndeezi, Juliet N. Babirye, Victoria Bakken, Ane-Marthe Solheim Skar, James K. Tumwine, Norbert Skokauskas

**Affiliations:** 1 Department of Psychiatry, School of Medicine, Makerere University College of Health Sciences, Kampala, Uganda; 2 Department of Psychiatry, Mulago National Referral and Teaching Hospital, Ministry of Health, Kampala, Uganda; 3 Centre for International Health, Department of Global Public Health and Primary Care, Faculty of Medicine, University of Bergen, Bergen, Norway; 4 Department of Pediatrics and Child Health, School of Medicine, Makerere University College of Health Sciences, Kampala, Uganda; 5 School of Public Health, College of Health Sciences, Makerere University, Kampala, Uganda; 6 Centre for Child and Adolescent Mental Health and Child Protection, Institute of Psychiatry, Faculty of Medicine, NTNU, Trondheim, Norway; 7 Global Health Cluster, Division for Health Services, Norwegian Institute of Public Health, Oslo, Norway; 8 Norwegian Centre for Violence and Traumatic Stress Studies, Oslo, Norway; 9 Department of Pediatrics and Child Health, School of Medicine, Kabale University, Kabale, Uganda

**Keywords:** Alcohol, CRAFFT tool, Other substance use, Primary school-age children (6 to 13 years), Screening, Uganda

## Abstract

**Background:**

Children at risk of substance use disorders (SUD) should be detected using brief structured tools for early intervention. This study sought to translate and adapt the Car, Relax, Alone, Forget, Family/Friends, Trouble (CRAFFT) tool to determine its diagnostic accuracy, and the optimum cut-point to identify substance use disorders (SUD) risk in Ugandan children aged 6 to 13 years.

**Methods:**

This was a sequential mixed-methods study conducted in two phases. In the first qualitative phase, in Kampala and Mbale, the clinician-administered CRAFFT tool version 2.1 was translated into the local Lumasaaba dialect and culturally adapted through focus group discussions (FGDs) and in-depth interviews, in collaboration with the tool’s authors. Expert reviews and translations by bilingual experts provided insights on linguistic comprehensibility and cultural appropriateness, while pilot testing with the target population evaluated the tool’s preliminary effectiveness. In the second phase, the CRAFFT tool, adapted to Lumasaaba, was quantitatively validated against the Mini International Neuropsychiatric Interview for Children and Adolescents (MINI-KID) for diagnosing SUD in Mbale district, through a survey. Participants, chosen randomly from schools stratified according to ownership, location, and school size, were assessed for the tool’s reliability and validity, including comparisons to the MINI KID as the Gold Standard for diagnosing SUD. Data were analyzed using STATA-15. Receiver-operating-characteristic analysis was performed to determine the sensitivity, specificity, and criterion validity of the CRAFFT with the MINI-KID.

**Results:**

Of the 470 children enrolled, 2.1% (n = 10) had missing data on key variables, leaving 460 for analysis. The median age and interquartile range (IQR) was 11 (9–12) years and 56.6% were girls. A total of 116 (25.2%) children had consumed alcohol in the last twelve-month period and 7 (1.5%) had used other substances. The mean CRAFFT score for all the children (n = 460) was 0.32 (SD 0.95). The prevalence of any alcohol use disorder (2 or more positive answers on the MINI KID) in the last 12 months was 7.2% (n = 32). The *Lumasaaba* version of the CRAFFT tool demonstrated good internal consistency (Cronbach’s α = 0.86) and inter-item correlation (Spearman correlation coefficient of 0.84 (p < 0.001). At a cut-off score of 1.00, the CRAFFT had optimal sensitivity (91%) and specificity (92%) (Area Under the Curve (AUC) 0.91; 95% CI 0.86–0.97) to screen for SUD. A total of 62 (13.5%) had CRAFFT scores of > 1.

**Conclusion:**

The *Lumasaaba* version of the CRAFFT tool has sufficient sensitivity and specificity to identify school-age children at risk of SUD.

**Supplementary Information:**

The online version contains supplementary material available at 10.1186/s13722-024-00465-7.

## Background

Uganda has been ranked among the top 10 countries in the world with the highest alcohol consumption [1, 2], and most of the alcohol consumed is unprocessed, unregulated, and therefore more dangerous [2]. With about half of its population (49.3%) under the age of 15 years [3], Uganda is home to one of the world’s largest populations of children. Studies have detected alcohol consumption by children in Uganda, with some starting as young as 5 to 8 years old [4–6]. In addition, we found that primary school-age children in Mbale district, Uganda, reported easy access to alcohol, consuming it in various settings, including their homes, schools, and even classrooms during instructional sessions [6]. Low family socioeconomic status has been linked to health inadequacies and behavioral risks, including the harmful consumption of alcohol [7], which is a concern given Uganda’s classification as a low-income country [8]. In this study, alcohol consumption by children was defined as the drinking of beverages containing ethyl alcohol, rather than just sips or tasting, among children n aged 6 to 13 years. Those children who drank alcohol or used other substances were described as “at risk children”.

Altho ugh alcohol use among young adolescents has been declining in recent years in some parts of the world [9], there is a growing burden of alcohol and other substance use among children and youth in most low- and middle-income countries (LMICs) [10, 11]. For example, a systematic review on substance use among adolescents (10–19 years) in sub-Saharan Africa, found a high prevalence of ‘any substance use’ at 41.6%, and alcohol use alone at 32.8% respectively [12]. In addition, researchers in Africa have reported onset for alcohol and other substance use starting even before the age of 11 years [13].

Alcohol consumption by children and adolescents often coexists with other substance use and mental health problems [10, 14], with major short and long-term effects for the users, their families, and society [15, 16]. For example, earlier researchers identified the early initiation of alcohol consumption before age 14 years as a significant predictor for later abuse and addiction [17–19]. It is therefore crucial for Uganda to prioritize the identification of children at risk of alcohol and other substance use for early intervention.

Routine screening for alcohol and other substance use among children is crucial to the early detection and prevention of problematic substance use and related harms. Earlier studies have shown the effectiveness of screening and referral for intervention and treatment for adolescents at risk of alcohol and substance use [20]. In line with this, the World Health Organization (WHO) recommends routine screening for substance use in adolescents by healthcare providers [21]. By using culturally adapted brief screening tools, healthcare providers can enhance the early detection of at-risk children [22, 23], which can lead to timely intervention and improved outcomes.

At the time of the study, there was a lack of concise and easy-to-use screening tools to detect alcohol and substance use in school-aged children in Uganda. This meant that the health system could potentially overlook at-risk children. Additionally, the Uganda Ministry of Health had limited data and reporting on alcohol and substance use among children through the health management information systems. Early screening aims to mitigate risks among children by detecting those who are abusing alcohol and other drugs at an early stage so that interventions can be implemented [24].

After reviewing multiple substance use screening tools for adolescents [25–27], we concluded that the Car, Relax, Alone, Forget, Family/Friends, Trouble (CRAFFT) tool [28] seemed most suitable for screening substance use risk among school-age children in Uganda. The CRAFFT tool has been previously validated in adolescent populations globally, demonstrating its effectiveness in identifying alcohol and substance use disorders [29]. It was found to be effective in identifying alcohol use disorders in primary care patients aged 12 to 17 years according to the Diagnostic and Statistical Manual of mental disorders fifth edition (DSM-5) [30], with a sensitivity of 0.79 and a specificity of 0.97 for identifying problem use or any DSM-5 alcohol use disorder (AUD) [31]. The tool is widely used in Africa and other parts of the world [32].

While initially developed for adolescents aged 12–18 years, we acknowledge the evolving landscape of substance use among children under 10 in Uganda [6], and the associated challenges in healthcare worker perceptions and age-appropriate screening methods. It is important to note that, at the time of our study, routine screening for substance use among primary school-age children, particularly those below 10 years, was not a standard practice in Uganda, yet there was evidence that children in this age group were engaging in alcohol consumption and other substance use. The brevity of the tool made it easy for clinicians to administer it in busy settings, and the statements could be easily understood by primary school-age children if well translated into the cultural context. Moreover, the CRAFFT tool provided estimates of the frequency, risk, and problematic alcohol and substance use, and its effectiveness had been validated in various contexts [24].

The utilization of the CRAFFT tool in our study for primary school-age children is rooted in the tool’s ability to capture early signs of substance-related risk. Our study seeks to contribute valuable insights into the effectiveness of the CRAFFT tool in identifying risk factors and guiding intervention strategies for this specific age group. In Uganda, many health workers in clinical practice do not ask about alcohol use in children mainly because they do not expect them to be taking it.

The aim of this study was to translate and adapt the CRAFFT tool into the Lumasaaba language and evaluate its psychometric properties in school-age children aged 6 to 13 years who were at risk for alcohol and other substance use disorders. It was part of the larger Child Alcohol Use Disorder (TREAT C-AUD) project in the Mbale district, Eastern Uganda [33], that followed the detection of alcohol use by young children [4]. Specifically, the study aimed to improve the tool’s ability to screen for alcohol and other substances for this vulnerable population in the Ugandan setting. In addition, the study is answering the question “Can the CRAFFT tool be used in children under 12 years?

## Methods

### Study design, aim, and setting

This sequential mixed-methods study employed both qualitative and quantitative approaches and was conducted in two phases. In the first phase, conducted in Kampala and Mbale, we qualitatively translated and adapted the clinician-administered CRAFFT tool version 2.1 into Lumasaaba, the local language for Mbale district in Eastern Uganda. This process involved engaging in focus group discussions (FGDs) and conducting in-depth interviews in collaboration with the authors of the CRAFFT tool. Expert reviews and translations by bilingual experts ensured linguistic clarity and cultural relevance. Subsequently, pilot testing with the target population was conducted to assess the preliminary effectiveness of the Lumasaaba adapted CRAFFT tool.

In the second phase, the CRAFFT tool, adapted to Lumasaaba, was quantitatively validated against the Mini International Neuropsychiatric Interview for Children and Adolescents (MINI-KID) for diagnosing SUD in Mbale district. This validation process involved a survey to determine the diagnostic accuracy and optimal cutoff score of the Lumasaaba CRAFFT tool in identifying children aged 6 to 13 years at risk of SUD. Participants were randomly selected from schools stratified by ownership, location, and size. The survey assessed the reliability and validity of the Lumasaaba CRAFFT tool, including comparisons to the MINI KID based on DSM-5 criteria, which served as the gold standard for diagnosing SUD. Data were collected between September 2019 and March 2021; the period was prolonged due to the country’s multiple lockdowns at the onset of the Covid-19 pandemic.

### Rationale for age range selection

In addition to the detection of alcohol use among children, we considered the primary school children age range normally expected at 6 to 12 years but we added another year in order to capture those who might start school late. Therefore, the choice of the age range (6 to 13 years) for the target population is informed by a dual consideration involving both the existing literature and our study objectives. While a substantial portion of international literature primarily addresses adolescents and older age groups concerning substance use, our study aimed to bridge a critical gap by focusing on the primary school-age population. This decision was influenced by the lack of comprehensive research on children under the age of 10, particularly in the context of substance use and related risk factors.

Furthermore, the pre-COVID protocol inclusion of primary school-aged children was intentional, driven by our objective to enhance awareness within school and health systems regarding this often-understudied group. As the largest school population, primary school children represent a crucial group for early intervention strategies and the development of targeted preventive measures.

Mbale district hosted the TREAT-CAUD project (33), which resulted from an earlier study that found clinically significant alcohol use by children [4]. At the time of data collection Mbale city was within the district and was categorized as urban while the town councils outside Mbale town were categorized as peri-urban. According to the Uganda National Bureau of Statistics, the population in Mbale was projected to be over 604,100 inhabitants by 2021 [34]. The school attendance percentage was 87% for boys and girls of primary school-age (6–12 years), and about one in ten primary school-aged children had never been to school. The dropout rate in primary schools in Mbale district was estimated at 8% per year [3]. *Lumasaaba* is the main language in Mbale but it has three main dialects, namely Lubuya, Lududa, and Ludadiri [35], of which Lubuya is the dialect that is most used in primary schools for the school curriculum. We decided to translate the CRAFFT tool into *Lumasaaba (Lubuya dialect)* and validate it for the target population of primary-school-age children (6 to 13 years) in Uganda.

### Ethical considerations

Ethical approval of the study protocol was obtained from the Makerere University School of Medicine Higher Degrees Research Ethics Committee (SOMREC # REC REF 2018-095), UNCST # SS5103 and Research Council of Norway West #107,632. Permission to translate and evaluate the CRAFFT was obtained from the authors of the CRAFFT tool [36]. In addition, permission to conduct the study was supported by the Mbale district administration and the head teachers of the respective schools. Informed consent from parents and assent from the child participants were also obtained. Teachers and parents were provided with information on the purpose of the study and our standard operating procedures had provisions for child protection. We prioritized the well-being and confidentiality of all participants throughout our research process. While we could not disclose specific details about individual participants, we ensured conditional confidentiality protocols were in place to protect the privacy of the participants who were identified as being at risk of alcohol use disorder. In cases where children scored high on the CRAFFT tool, the team lead (with special training in child and adolescent psychiatry and mental health, worked closely with their parents or guardians to provide appropriate support and guidance. This included offering reassurance, resources, referrals to relevant healthcare professionals or support services, and facilitating open communication between the parents, and healthcare providers. Our study team remained actively engaged with school communities throughout the screening process, offering opportunities for dialogue and addressing any concerns or questions that arose, and providing guidance on how to support children who may be at risk.

### The CRAFFT substance use screening tool

The original CRAFFT tool is a 6-item, developmentally appropriate clinical screening tool that was developed by Knight et al. [28, 36, 37] to screen for substance-related risks and problems in adolescents aged 12 to 18 years in the United States. When administered in a primary health care setting, the CRAFFT had good discriminative properties for determining alcohol and other substance use disorders in adolescents, with a high sensitivity (0.97) and specificity (0.80) [37]. It can be used in two ways: adolescents between the ages 12–18 years can complete it themselves or it can be administered by a clinician. In this study, we translated, adapted and validated the clinician administered version 2.1 of the CRAFFT tool. This version comprises two sections A and B. Section A has three items to evaluate the frequency of use of alcohol, marijuana, and other substances within the past 12 months period. Section B comprises of the six items (CRAFFT) that evaluate the risk (Car) and problem use (Relax, Alone, Forget, Family/Friends, Trouble) [38]. If the adolescent answers 0 for all the 3 frequency questions in section A, the clinician asks only the “Car” question and stops. If the adolescent has 1 or more scores for any of the 3 frequency questions in section A, then the clinician asks all the CRAFFT items in section B. Each “Yes” response on the CRAFFT is scored 1, giving a total score of 6. The adolescent is considered to be at low risk if there is no use of alcohol, marijuana, or other drugs in the past 12 months and the “Car” question is negative. Medium risk is when there is any past 12 months use plus a score of 0 to 1 on the CRAFFT tool while the high risk is any past 12 months use plus a score of 2 or higher.

The mnemonic of the CRAFFT (section B of the questionnaire) is as follows:

1. Have you ever ridden in a CAR driven by someone who was high or had been using
  Drugs or alcohol?
2. Do you ever use alcohol or drugs to RELAX, feel better about yourself, or fit in?
3. Do you ever use drugs or alcohol when you are ALONE?
4. Do you FORGET things you did while using drugs or alcohol?
5. Does your FAMILY or FRIENDS ever tell you that you should cut down your drinking or.drug use?
6. Have you ever gotten into TROUBLE while using drugs or alcohol?

## Phase 1 Translation and cultural adaptation of the CRAFFT tool

### Procedure

We translated and adapted the clinician administered version of the CRAFFT screening tool following the World Health Organization (WHO) guidelines for translation and adaption of instruments (40), and the simplified version by Sousa, V. D. et al. [39]. The process involved collaboration with the authors of the CRAFFT tool 2.1 from the Center for Adolescent substance abuse Research (CeASAR) [37].

An expert multidisciplinary committee comprising of 13 members was constituted (a child and adolescent psychiatrist, 2 pediatricians, 2 clinical psychologists, 2 social workers, 2 primary school teachers, 2 psychiatric clinical officers, and 2 pediatric nurses). These reviewed the idiomatic, semantic, cultural, and conceptual aspects of the CRAFFT tool and assessed the statements for easy comprehension of the items by Ugandan children ages 6 to 13 years. Forward translations of the CRAFFT tool from English to *Lumasaaba* language were conducted by two independent bilingual translators whose native language was *Lumasaaba*. The translated version was back-translated to English by two different independent translators. The back-translated version was reviewed by the multidisciplinary expert committee together with the translators, and the conciliated version was shared with the authors of the CRAFFT tool to produce a prefinal version. The prefinal version of the *Lumasaaba* CRAFFT tool was then piloted among 47 primary-school-age children aged 6 to 13 years considering age and gender as well as rural, peri-urban, and urban areas. For each statement in the CRAFFT tool, the participants were asked to give their views on how they felt when asked the question; what they thought the statement was asking about; whether there were any words that were difficult to understand or annoying; if the statement made them feel like taking an alcoholic drink or use any other substances and if yes, how likely that they would do so. The feedback from the children was reviewed by the committee in collaboration with the authors of the CRAFFT tool. The proposed changes were applied to produce the final Lumasaaba CRAFFT tool.

### Results of the adaptation process

We found that the clinical officers and nurses had challenges interpreting the instructions for clinicians when administering the CRAFFT tool. Therefore we added statements overleaf to explain conditional confidentiality, the twelve months period and how to fill the boxes in part A, and how to proceed to part B of the CRAFFT tool.

During the pretest some children reported feeling bad when they were asked about alcohol or other substances yet they did not drink. To mitigate this emotional discomfort, we incorporated an opening statement in the Lumasaaba CRAFFT tool designed to prepare the child for the forthcoming discussion. Following initial greetings and self-introduction, the interviewer would explicitly state, "I want us to talk about alcohol, marijuana, and other drugs." This statement aimed to establish a transparent and non-threatening context for the conversation. To further gauge the child’s comfort level, two YES-or-NO questions were integrated into the introductory phase. Specifically, the child was asked if they had ever heard about alcohol and if they had friends who consumed alcohol. If the responses to both questions were negative (NO), the assessment would not proceed with the detailed questioning on this topic, respecting the child’s boundaries. Where we detected discomfort we re-assured the children and further explained the purpose of the study and conditional confidentiality.

It is noteworthy that during these discussions, we observed positive non-verbal cues from some children, such as smiles or lip movement and many children had no problems with the questions. While these expressions may suggest a level of engagement or ease for some participants, we acknowledge that individual reactions can vary.

We found some idioms which were not familiar to children in Uganda such as “vaping”, “K2”, “Spice”, and were dropped. The words which children reported as difficult such as “high”, “relax”, “trouble” were interpreted. Alcoholic beverages and other substances that were easily identified by children like “locally made beer”, “marijuana cookies or sweets”, “gum”, “nail varnish”, “airplane/aviation fuel” were added. The CAR question was revised and substituted with Boda-Boda (bicycles, motorcycles and scooters) as these were found relevant for this age group in the Ugandan context, however the acronym CRAFFT was maintained. Additional file 1 Table showing items in the original CRAFFT tool, adjustments and the final *Lumasaaba* version. After completing the translation and adaptation of the clinician-administered CRAFFT tool into Lumasaaba, the final version was shared with the authors of the CRAFFT tool for review. The authors reviewed the Lumasaaba version and provided their approval, indicating that it met the necessary criteria for acceptance. Additional file 2 The Lumasaaba version of the clinician administered version of the CRAFFT tool.

## Phase 2 Psychometric testing of the final *Lumasaaba* CRAFFT tool

### Participants and sampling

The target population was all school-age children (6 to 13 years old) residing in Mbale district. The source population was all school-age children attending public and private schools within Mbale district. A list of 176 schools in total was obtained from the planning unit of Mbale district (January 2019). Schools were stratified by ownership (public or private), by school categories as per social economic status (deciles) i.e. rural, urban or peri-urban, and school size. Deciles, from 1 to 10 roughly indicate the socioeconomic characteristics of the families in the school zone, were grouped into different levels: rural (1 and 2), peri-urban (3 to 8), and urban (9 and 10). Forty-six schools were selected using systematic sampling without replacement for each decile band. Of the forty-six head teachers contacted 38 accepted for their schools to participate in the study.

The study sample was primary school children (grades 1 to 7) who were randomly selected from the thirty-eight schools in Mbale district. The sample was determined using ‘Yamane’s method [40]. The number of students/pupils sampled from each stratum was proportional to the number of schools in each stratum. The selection of study participants was achieved by computer-generated random numbers using the class lists. This gave equal probability for all participants for study enrollment.

The study participants were identified through their schools, located either in rural, urban, or peri-urban areas of the district. The inclusion criteria were: children in the age group 6 to 13 years, studying in Mbale district, in schools where the head teachers gave permission for their schools to participate in the study, the parents gave informed consent and the children themselves assented to participate in the study. Children who self-reported as ill at the time of data collection (number not known) were excluded from the study.

With a level of precision at 5%, 400 participants was the target sample size. To account for possible missing data and to increase the power of this study to answer all the objectives, the sample size was increased by 20% giving a total of 480. We contacted all parents of the 480 sampled children and all of them consented for their children to participate in the study. However, at the time of data collection due to delays following Covid 19 country lockdown, some participants were no longer eligible to participate in the study, some children had turned 14 years, and other girls had dropped out of school due to pregnancy, leaving a total of 470 participants. Additional file 3: Fig. S1 Flow diagram showing the sampling of schools and study participants.

### Data collection measures

#### The Lumasaaba version of the CRAFFT

The Lumasaaba CRAFFT requires that the clinician introduces the topic of discussion and explains the meaning of conditional confidentiality to the patient. The tool also has two screening Yes/No questions asking about whether the child has heard about alcohol and if they have friends who drink alcohol. If the child the response is NO to both questions then the clinician does not proceed with the entire interview.

Part A.

Part A requires the clinician to explain the meaning of the 12 months period, before asking the frequency questions, and to record the number of days that the child reports to have drunk alcohol or used marijuana or any other substances during the past 12 months period. Part B asks the risk and problem substance use questions and requires the clinician to explain the meanings of the words as indicated in the respective statements. At the end of the questionnaire the is a foot note (* Any use of alcohol or any drug whether or not they give any YES answers is worrisome in younger children and indicates need for further assessment.*) and maintaining the copyright for the author of the CRAFFT tool. Additional file 2 The Lumasaaba version of the clinician administered version of the CRAFFT tool.

#### The Mini International Neuropsychiatric Interview for Children and Adolescents for DSM 5 (MINI KID)

The MINI KID, developed by Sheehan et al. [41], is a structured clinical diagnostic interview that enables researchers to make diagnoses of psychiatric disorders in children and adolescents, including alcohol and other substance use disorders, according to the DSM-5 [30]. The tool has various diagnostic questions for each module, with two screening questions corresponding to the main criteria for the specific DSM-5 disorders. The questions are designed in a way that makes them to be easily understood by children and adolescents. If the screening questions are negative, there is no need to ask for additional symptoms. In this way, the tool can be easily and quickly administered by trained personnel. The authors permits researchers to use paper copies of the tool for use in nonprofit or publicly owned settings [41].

The MINI KID was validated and found to have good psychometric properties for diagnosing child and adolescent psychiatric disorders with the area under the curve (AUC) (0.81–0.96, kappa = 0.56–0.87) when compared to the Schedule for Affective Disorders and Schizophrenia for School-Age Children-Present and Lifetime version (K-SADS-PL) . Although the psychometric properties of the MINI KID have not yet been determined in Uganda, it has been used in various studies [42–44]. The alcohol use disorder module in the MINI KID has screening questions to decide if a child or adolescent has had 3 or more drinks in a day, if they had 3 or more drinks in 3 h and if this happened 3 or more times in the past year. The additional 11 symptoms are asked if the participant responds with a “yes” to all the screening questions, for the clinician to explore for alcohol use disorder in the past 12 months and to determine its severity. For a diagnosis of alcohol use disorder (AUD), one has to meet two or more of the eleven criteria during the same 12 months period. The severity depends on the number of criteria met, mild (2–3), moderate (4–5), and severe (6 or more).

For the substance use disorder (non-alcohol) module, the clinician reads through a list of drugs or medicines. The participant is expected to indicate if they have used any of them more than one time in the past year either to get high or change their mood. For a diagnosis of substance use disorder (non-alcohol), one has to meet 2 or more of the 9 diagnostic criteria for use within the same 12 months period.

In this study, we utilized the alcohol use disorder and substance use disorder modules of the MINI KID, which is widely considered to be the gold standard for clinical diagnosis of alcohol use disorder and other substance use disorders in children when administered by an experienced clinician.

### Data collection procedure

Prior to the day of data collection, parents of the selected child participants were approached and invited with the help of the respective school administration. The child participants were subjected to semi structured clinical interviews using a social demographic questionnaire, the Lumasaaba version of CRAFFT tool and the MINI KID. The assessments were conducted by experienced clinicians; JSN who is a psychiatrist with special training in child and adolescent psychiatry, and IA and NM who are experienced psychiatric clinical officers.

### Effects of COVID-19

Following the country lockdown due to the COVID-19 pandemic, all schools in Uganda were closed in March 2020. This caused delays in data collection and affected easy access to the children. This caused several challenges, such as extra costs of tracing pupils as some had already moved from their homes, some could not be traced as their parents had changed contact information, and some girls had either married or become pregnant. Furthermore, some previously trained research assistants had already moved to other jobs. We recruited and trained five additional research assistants and worked with the schools to mobilize the pupils from their homes by phone and by home visiting using people who knew them. Finally, we collected data following standard procedures for COVID-19 infection prevention and control set by the Uganda Ministry of Health and the Uganda National Council for Science and Technology (UNCST).

### Statistical analysis

Participants’ socio-demographic characteristics and the diagnostic classifications were computed by calculating frequencies and percentages. Affirmative responses to the CRAFFT questionnaire were described using frequencies and percentages. Gender comparisons on the affirmative responses to the CRAFFT tool were computed using Fischer’s exact tests. A composite score was generated as a summation of the 6-items of the CRAFFT tool. Complete case analysis was considered during analysis. This included dropping the few observations that were missing on the very important variables of interest.

Internal consistence of the *Lumasaaba* version of the CRAFFT tool was assessed using Cronbach’s alpha coefficient. Inter-item correlations for the 6 CRAFFT items was evaluated using the Spearman correlation coefficient. The correlation coefficient was assessed between the CRAFFT tool items and diagnostic classifications for substance use on the alcohol and substance use disorder modules of the MINI KID using the spearman’s correlation. Measurement of invariance analysis was not done because the study was cross-sectional. Criterion validity was done with the alcohol use disorder and substance use disorder modules of the MINI KID as the Gold Standard.

Six cut-off points were generated from the 6 items of the CRAFFT tool. For each cut-off point, sensitivity and specificity analyses were done with the clinician administered MINI KID as “the gold standard”. The cut-off points were 6 to represent each of the 6 items of the CRAFFT tool. For each of the 6 screening questions, sensitivity analysis was done until an optimal point was reached.

We analyzed the Lumasaaba CRAFFT test to determine the best score to diagnose SUDs in children. We conducted receiver operating characteristic curves analysis to determine the optimal cut-off point for the Lumasaaba CRAFFT test, its sensitivity, specificity, positive predictive value, and negative predictive value [45], and compared to MINI KID at CRAFFT scores from 1 to 6. We calculated the Area Under Curve to measure the accuracy of the Lumasaaba CRAFFT test and the 95% confidence intervals to ensure reliability of our findings.

## Results

### Participant characteristics and substance use patterns

Of the 470 school children who participated in the study, data for 10 (2.1%) participants was dropped due to missing on key variables leaving data from 460 participants for the analysis. Participants were 56.5% (n = 260) girls, majority were Bagishu (Bamasaaba) (338, 73.8%). The sample was divided according to age range 6 to 9 years and 10 to 13 years. Over two-thirds 70.9% (n = 320) of the respondents were aged between 10 to 13 years, the median (IQR) age was 11 (9 to 12) years, and 60% (n = 276) were in the lower primary school classes P1-P4. More than half of the participants 65.4% (n = 301) were staying with both biological parents, while the rest stayed with other relatives. All the participants had a religious affiliation where the majority were Christians (Anglicans (29.5%), Roman Catholics (14.8%), Pentecostals (18.6%), Seventh Day Adventists (1.5%)), and the rest were Muslims.

Majority of participants 86.9% (n = 398) were enrolled in government schools while 13.1% (n = 62) were in private schools. Regarding school location, 55.6% (n = 256) of the schools were situated in urban or peri-urban areas, while 44.4% (n = 204) hailed from rural settings. Family structures varied, with 9.1% (n = 42) residing with both parents, 82.6% (n = 380) with a single biological parent, and 8.3% (n = 38) with others. Parental education levels exhibited a distribution across primary (51.3%, n = 234), secondary (28.1%, n = 128), and tertiary (20.6%, n = 94) categories. In terms of the source of parents’ income, 12.4% (n = 57) were derived from formal employment, 76.9% (n = 354) from informal employment, and 10.6% (n = 49) from other sources.

Table 1 shows the sociodemographic characteristics of the participants and the frequency of alcohol use.

**Table 1:** Sociodemographic characteristics of the participants and frequency of alcohol use in the past 12 months identified by the *Lumasaaba* CRAFFT tool

Factor	Numbers of participants, N = 460 (%)	Frequency of alcohol use *Lumasaaba* CRAFFT	
		N = 115 (%)	95% Confidence interval of the proportions
Sex			
Boys	200 (43.5)	49 (42.6)	(38.9: 48.1)
Girls	260 (56.5)	66 (57.4)	(51.8: 61.1)
Age			
6 to 9 years	131(29.1)	22 (19.6)	(16.3: 22.1)
10 to 13 years	320 (70.9)	90 (80.4)	(76.9:84.1)
Class level			
P1–P4	276 (60.0)	69 (60.0)	(56.1: 64.1)
P6–P7	184 (40.0)	46 (40.0)	(36.1: 43.9)
Religion			
Christian	295 (64.4)	100 (87.7)	(84.3: 90.2)
Moslem	163 (35.6)	14 (12.3)	(10.3: 14.3)
School ownership			
Government	398 (86.9)	109 (94.8)	(91.5: 97.6)
Private	62 (13.1)	6 (5.2)	(2.1: 8.2)
School location			
Urban/Peri-Urban	256 (55.6)	48 (41.7)	(38.7: 44.7)
Rural	204 (44.4)	67 (58.3)	(55.3: 61.4)
Family (who child stays with)			
Both parents	42 (9.1)	22 (19.1)	(15.1: 23.2)
Single biological parent	380 (82.6)	85 (73.9)	(70.9: 77.8)
Other	38 (8.3)	8 (6.9)	(5.8: 8.1)
Parents education			
Primary	234 (51.3)	70 (60.9)	(56.1: 64.2)
Secondary	128 (28.1)	31 (26.9)	(22.9: 29.4)
Tertiary	94 (20.6)	14 (12.2)	(9.6: 15.3)
Parents’ source of income			
Formal employment	57 (12.4)	10 (8.7)	(6.4: 10.3)
Informal employment	354 (76.9)	95 (82.6)	(80.6: 84.6)
Others	49 (10.6)	10 (8.7)	(6.7: 10.7)

According to the CRAFFT tool, more than half of the participants, 58.1% (n = 268), had heard about alcohol but had no friends using alcohol. Out of the total number of participants who reported using alcohol and/or other substances during the past 12 months period, a total of 118 (25.6%) reported using alcohol and/or other substances, 115 (25.0%) used alcohol only, 7 (1.5%) reported using marijuana or other substances not alcohol, while 3 (0.7%) reported using both alcohol and other substances. Table 1 shows the sociodemographic characteristics of the participants and the frequency of alcohol use (any affirmative response to the CARFFT in the last 12 months).

A total of 81 (17.6%) participants reported using alcohol or other substances but did not meet any criteria on the AUD module of the MINI KID, which suggests that they were experiencing problem use. Out of those who fulfilled the criteria for any AUD 32 (7.5%), 18 (4.0%) had mild, 4 (0.9%) had moderate, and 10 (2.3%) had severe AUD. Among the seven children who reported using substances other than alcohol, none met the criteria for SUD as defined by the MIN KID substance use (non-alcohol) module.

Of those who met the criteria for any alcohol or substance use, 25 (17.0%) were in the age group 6–9 years, while 90 (28.1%) were in the 10-to-13-year age category, girls were 66 (57.4%). Ten respondents (2.2%), had an affirmative response to the “Car” question. Of those having used alcohol or other substances in the past twelve months (n = 118), 40% (n = 48) gave affirmative responses to drinking alcohol or using other drugs to relax, 15.8% (n = 19) had drunk when they were alone, 9.3% (n = 23) reported to have forgotten things they did while being drunk, 26.9% (n = 32) reported having been advised by family or friends to stop drinking alcohol, while 12.7% (n = 15) had been in trouble because of drinking alcohol or using other drugs. It should be noted that a participant would give affirmative responses to more than one CRAFFT item.

### Internal consistency reliability of the CRAFFT tool

Cronbach’s alpha for the 6-item *Lumasaaba* version of the CRAFFT in this sample was 0.86. Spearman’s correlation coefficient indicated a moderate positive inter-item correlation among the 6 items of the CRAFFT tool. The “Car” question and “Relax” items were least correlated while “Relax, Alone, Forget, Family, and Trouble were most inter-correlated (Table 2).

**Table 2:** *Lumasaaba* version of the CRAFFT: inter-item correlation

	Car	Relax	Alone	Forget	Family	Trouble
Car	1.00					
Relax	0.13	1.00				
Alone	0.18	0.43^a^	1.00			
Forget	0.14	0.47^a^	0.33^a^	1.00		
Family	0.21	0.58^a^	0.33^a^	0.48^a^	1.00	
Trouble	0.21	0.47^a^	0.40^a^	0.52^a^	0.52^a^	1.00

^a^*A Significant pairwise correlation at a 5% level of significance. Spearman rho, 0.84:p* < *0.001*

Spearman’s rho coefficient was used to determine the extent to which items on the scale are assessing the same content. It was used to provide an assessment of item redundancy on the scale. The results showed moderate correlations between each other

### Criterion validity of the CRAFFT tool

The CRAFFT score correlated strongly with the MINI KID diagnostic classification (Spearman’s rho 0.62; p-value < 0.001).

Table 3 shows the sensitivity and specificity of the CRAFFT tool at different cut-off points in detecting substance use disorder compared to MINI KID.

**Table 3:** Sensitivity, Specificity, Positive and Negative Predictive Values of the *Lumasaaba* version of the CRAFFT tool

CRAFFT Score	Sensitivity	Specificity	Predictive values		% of cases correctly classified	AUC (95%CI)
			Positive	Negative		
≥ 1^*^	0.91	0.92	0.47	0.99	0.92	0.92 (0.86; 0.97)
≥ 2	0.75	0.96	0.60	0.98	0.94	0.87 (0.78; 0.93)
≥ 3	0.53	0.98	0.72	0.96	0.95	0.76 (0.69; 0.86)
≥ 4	0.34	0.99	0.78	0.95	0.94	0.67 (0.58; 0.75)
≥ 5	0.15	0.99	0.83	0.93	0.94	0.58 (0.51; 0.64)
6	0.03	1	1	0.93	0.93	0.52 (0.48; 0.54)

Asterisk (*) indicates the optimal cut point. (i.e. the maximum product of sensitivity and specificity, and different cut-off points in identifying any alcohol or other substance use disorder ascertained by the MINI KID. The cut-off score for the CRAFFT tool was found to be ≥ 1 with a sensitivity, specificity, and percentage correctly classified of 0.91, 0.92, and 0.92 respectively

### Optimal CRAFFT cut point

**Fig. 1:**
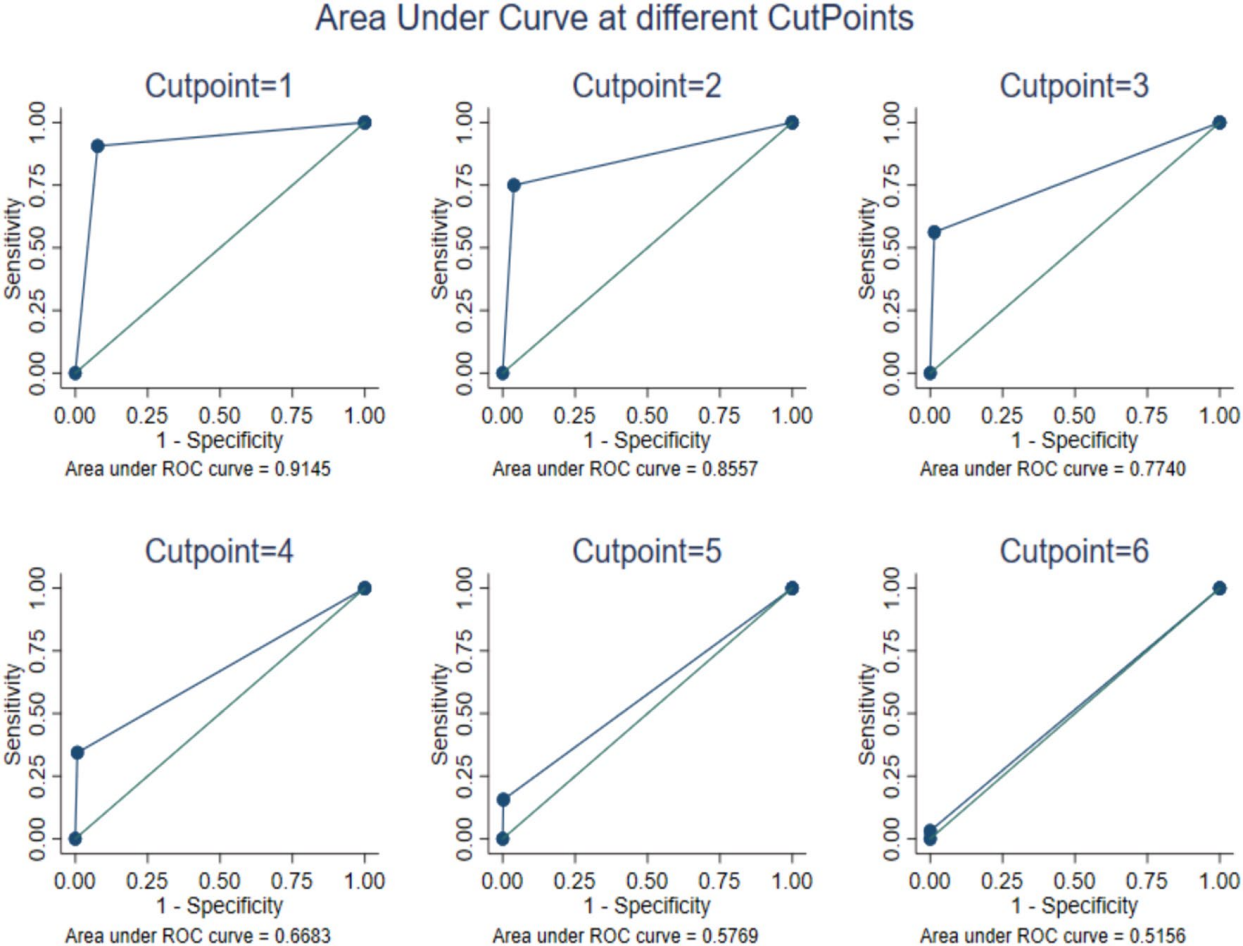
Area under the curve at different cut-off points

Receiver operating characteristic curves (ROCs) are presented in Fig. 1. The curves plot sensitivity against 1-specificity, such that the area under the curve is a measure of the CRAFFT tool’s screening accuracy. The optimal cut-point was selected based on the maximum product of sensitivity and specificity. At cut point 1, sensitivity was 0.91 while specificity was 0.92 giving a product of 0.84. The area under the curve for detecting alcohol or other substance use disorders was almost similar for boys (0.91) and girls (0.92). Using a cut-off score of 1, there was a consistent reduction in AUC with increasing age (Table 4). The CRAFFT had good discriminatory power for subjects with alcohol or substance use disorders (n = 29) as confirmed by the MINI KID (N = 446), with an area undercurve of 0.91.

**Table 4:** Psychometric properties of the CRAFFT tool (Overall, Sex and Age)

Overall	Sensitivity (%)	Specificity (%)	PPV (%)	NPV (%)	ROC curve
	90.6	92.3	47.5	99.2	91.4
Sex					
Boys	86.7	94.9	59.1	98.8	90.8
Girls	94.1	90.2	41.0	95	92.1
Age (years)					
6 to 7	100	97.1	50	100	98.6
8 to9	100	95.4	20	100	97.7
10 to 11	100	90.8	42.1	100	95.4
12 to 13	86.4	90.1	54.3	97.9	88.2

"Optimal cut-off" is the value for which the point on the ROC curve has the minimum distance to the upper left corner (where sensitivity = *1 and specificity* = 1). By Pythagoras’ theorem this distance is sqrt (1-sensitivity) ^2^ + (1-specificity) ^2^)

Receiver operating characteristic (ROC) curve at different cut-off points of the Lumasaaba version of the CRAFFT substance abuse screening tool for detection of alcohol or other substance use disorders among primary school-age children in eastern Uganda.

## Discussion

This study represents the first known attempt to validate the CRAFFT tool in a community sample of primary school-age children (6 to 13 years). The findings provide evidence to support the reliability of the Lumasaaba version of the CRAFFT for identifying substance use risk and problem use among children. In our study, a cut-off score of 1 or higher demonstrated optimal sensitivity and specificity for screening substance use risk and disorders in primary school-aged children in Uganda.

Substance use disorders among children and adolescents often go unnoticed, leading to limited access to care and treatment [22]. Early initiation of alcohol use has been linked to an increased risk of developing alcohol use disorders and other health-related complications later in life [46]. Timely identification of substance use, including alcohol, among school-aged children can play a vital role in primary health care to prevent the development of substance use and identify the need for brief interventions [47]. Given the limited resources in most LMICs, such as Uganda, task shifting may be necessary to address this issue [48]. Therefore, early identification of substance use among school-aged children could be especially beneficial in LMICs to improve the quality of life for affected children and their families, and the prevention of substance use disorders, which can be challenging and costly to treat later in life.

The CRAFFT tool was initially validated among 14–18-year-old clinic-attending patients in the United States [36], and subsequently validated among adolescents in various settings [37, 49]. The tool was validated for use among 12–18 year-olds in Germany [50], and the same age group for the Korean and Spanish versions [51, 52], while the Nigeria version included 13 to 17-year-old adolescents [53]. Across all these studies, the CRAFFT tool demonstrated good performance as a screening tool for alcohol and other substances among adolescents in different cultural and societal settings [29].

Our study found that the CRAFFT tool performed well in screening for substance risk and problem use among children aged 6 to 13 years, with better performance observed in younger children compared to older children. This finding is supported by a consistent decrease in diagnostic accuracy, as measured by the area under the curve (AUC), with increasing age. The AUC is a widely used measure of the overall diagnostic accuracy of a screening tool, with values closer to one indicating higher accuracy [54]. Therefore, our study provides evidence for the potential usefulness of the CRAFFT tool in identifying substance use risk and problem use in children aged 6 to 13 years. Although our study provides evidence for the potential usefulness of the CRAFFT tool in accurately identifying substance use among young children, it is important to note that our findings have not been compared to those of previous studies as we did not find any validation studies of the CRAFFT tool among children aged 6 to 13 years.

In terms of sex, our study found no significant differences in the performance of the CRAFFT tool in identifying substance use risk and problem use among boys and girls. This is consistent with previous studies that have validated the tool among adolescents of both sexes [28, 37, 50–52]. This implies that the CRAFFT tool is not biased towards one gender and is equally effective in identifying substance use problems among all children, regardless of their sex.

Our study found that the *Lumasaaba* version of the CRAFFT tool has good internal consistency reliability for screening substance use among Ugandan primary school-aged children, with a Cronbach’s alpha of 0.86. This is consistent with previous studies, which have reported Cronbach’s alpha values ranging from 0.65 to 0.86 for the CRAFFT tool [29]. Recent studies on the Spanish and Nigerian versions of the tool found similar reliability with Cronbach’s alpha values of 0.75 and 0.85, respectively [52, 53]. Overall, these findings support the use of the Lumasaaba CRAFFT tool as a reliable screening tool for substance use among Ugandan school-aged children population.

The optimal cut-off score for the CRAFFT tool in screening for substance use was found to be 1 or higher in this study, with sensitivity and specificity values of 0.91 and 0.92 respectively. This indicates that the tool accurately identified those with and without alcohol or other substance use disorders 91% and 92% of the time, respectively. Similar results were observed in the Singapore version for the multiethnic Asian population of young males [55]. However, other studies have suggested different cut-off scores for different settings and age groups ranging from one or more to two or more [52, 56]. Given the young age group in our study and the importance of child safety, we recommend using a cut-off score of 1 or higher to prevent missing an opportunity to detect a child at risk of substance use or disorder. Any child with a positive response to any of the CRAFFT items should undergo further clinical assessment for prevention and early intervention.

### Utility

Our study indicates the potential value of the CRAFFT tool for healthcare providers in identifying at-risk children and implementing interventions in the studied region of Uganda. While the study focused on a specific region in Uganda, it is crucial to acknowledge the need for caution in generalizing the results to the entire country. However, on the whole, alcohol consumption in Uganda is quite worrying [5, 6, 57]. As such, use of a simple tool like the one we have adapted would be a great resource and strength in the identification of affected children. We recognize the importance of conducting further studies across diverse regions to enhance the external validity of our findings.

### Strengths and limitations

The study is the first to validate the CRAFFT tool among school-aged children, which is a significant contribution to the literature. The study used a gold standard diagnostic tool, the MINI KID, to compare the CRAFFT scores, which increases the reliability of the findings. The sample size of 460 participants enhanced the statistical power of the analysis, and the structured clinical interviews were held by experienced psychiatrists and clinical officers, which enhanced the validity of the data. However, this study has some limitations that warrant consideration when interpreting the findings. Firstly, we only included school-attending children in the sample. This may have resulted in the exclusion of primary school-aged children who are not enrolled in formal education systems, thereby potentially limiting the generalizability of our findings to the broader population of primary school-aged children in the area. However, Uganda has a universal primary education policy and most (91%) of the primary school-age children in the region attend school [3, 58]. Secondly, data was collected using interviews and questionnaires which might have introduced recall and social-desirability bias [59]. Furthermore, the study was cross-sectional and test–retest reliability was not assessed. Even though the psychometric properties of the MINI KID had not been assessed in Uganda, this instrument has been used in numerous studies targeting child and adolescent mental disorders in other contexts [42, 43]. In particular, it was validated and found to be useful in specialized child and adolescent psychiatric outpatient diagnostic processes in a Nordic (Sweden) context [60]. Finally, while strategic measures, such as incorporating an opening statement designed to prepare the child for the forthcoming discussion, and utilizing YES-or-NO questions in the Lumasaaba CRAFFT tool, aim to create a considerate approach in addressing the sensitivity of discussing alcohol and drugs among children, it is recognized that these efforts may not entirely eliminate discomfort. Nevertheless, these methods contribute to identifying children at risk of substance use early in the assessment process for timely intervention and support. It is worth noting that further research may be necessary to assess the validity of the Lumasaaba CRAFFT for use with older adolescents.

## Conclusion

Our study found that the *Lumasaaba* version of the clinician-administered CRAFFT tool is useful for identifying school-age children at risk of substance use, with sufficient sensitivity and specificity. The CRAFFT tool can be implemented in health and school settings to improve the identification of high-risk children and to refer them for further assessments and care. We recommend that the Lumasaaba CRAFFT tool be translated into other languages to enable screening for alcohol and substance abuse in other regions of the country. The use of the CRAFFT tool could also improve the generation of data on the burden of alcohol and substance use in school-age children, which can inform better planning for prevention and treatment interventions. The findings may guide further research, and early intervention strategies for school-age populations in low- and middle-income countries, where substance use is a growing public health concern.

### Supplementary Information

**Additional file 1: **Table showing items in the original CRAFFT tool, adjustments and the final Lumasaaba version.

**Additional file 2 : **The Lumasaaba version of the clinician administered version of the CRAFFT tool.

**Additional file 3 : **Flow diagram showing the sampling of schools and study participants.

## Data Availability

The datasets used and/or analyzed during the current study are available from the corresponding author on reasonable request.

## Abbreviations

AUD: Alcohol use disorder
AUC: Area under the curves
CRAFFT: Car, Relax, Alone, Forget, Family/Friends, Trouble
DSM 5: Diagnostic and statistical manual of mental disorders fifth edition
LIC: Low-income country
LMIC: Low- and middle-income country
MINI KID: The Mini International Neuropsychiatric Interview for Children and Adolescents for DSM 5
NPV: Negative predictive value
PPV: Positive predictive value
ROC: Receiver operating characteristic curve
SUD: Substance use disorder
SSA: Sub-Saharan Africa

## References

[1] World Health Organization. Management of substance abuse unit. Global status report on alcohol and health. 2014.

[2] World Health Organization. Global status report on alcohol and health 2018: World Health Organization. 2019.

[3] Uganda Bureau of statistics. The national population and housing census 2014-National analytical report. 2017.

[4] Engebretsen IMS, Nalugya JS, Skylstad V, Ndeezi G, Akol A, Babirye JN (2020). "I feel good when I drink"-detecting childhood-onset alcohol abuse and dependence in a Ugandan community trial cohort. Child Adolesc Psychiatry Ment Health.

[5] Skylstad V, Nalugya J, Skar A-MS, Opesen C, Ndeezi G, Okello E (2022). ‘As soon as they can hold a glass, they begin taking alcohol’: a qualitative study on early childhood substance use in Mbale district Uganda. BMC Public Health.

[6] Nalugya JS, Skylstad V, Babirye JN, Ssemata AS, Ndeezi G, Bangirana P (2023). "She gives it to her child who doesn’t even talk": a qualitative exploration of alcohol and drug use among primary school-age children in Uganda. BMC Public Health.

[7] Huq T, Alexander EC, Manikam L, Jokinen T, Patil P, Benjumea D (2021). A systematic review of household and family alcohol use and childhood neurodevelopmental outcomes in low- and middle-income countries. Child Psychiatry Hum Dev.

[8] Countries and economies. 2022. https://data.worldbank.org/country. Accessed 7 April 2023

[9] Kraus L, Room R, Livingston M, Pennay A, Holmes J, Törrönen J (2020). Long waves of consumption or a unique social generation? Exploring recent declines in youth drinking. Addictn Res Theory.

[10] Erskine H, Moffitt TE, Copeland W, Costello E, Ferrari A, Patton G (2015). A heavy burden on young minds: the global burden of mental and substance use disorders in children and youth. Psychol Med.

[11] Degenhardt L, Stockings E, Patton G, Hall WD, Lynskey M (2016). The increasing global health priority of substance use in young people. The Lancet Psychiatry.

[12] Ogundipe O, Amoo E, Adeloye D, Olawole-Isaac A (2018). Substance use among adolescents in sub-Saharan Africa: a systematic review and meta-analysis. S Afr J Child Health.

[13] Ndetei DM, Khasakhala LI, Mutiso V, Ongecha-Owuor FA, Kokonya DA (2009). Patterns of drug abuse in public secondary schools in Kenya. Subst Abuse.

[14] Ganz D, Sher L (2009). Suicidal behavior in adolescents with comorbid depression and alcohol abuse. Minerva Pediatr.

[15] Park SH, Kim DJ (2020). Global and regional impacts of alcohol use on public health: Emphasis on alcohol policies. Clin Mol Hepatol.

[16] Babor TF, Babor T (2010). Alcohol: no ordinary commodity: research and public policy.

[17] DeWit DJ, Adlaf EM, Offord DR, Ogborne AC (2000). Age at first alcohol use: a risk factor for the development of alcohol disorders. Am J Psychiatry.

[18] Hingson RW, Heeren T, Winter MR (2006). Age at drinking onset and alcohol dependence: age at onset, duration, and severity. Arch Pediatr Adolesc Med.

[19] Miller MW, Spear LP (2006). The alcoholism generator. Alcohol Clin Exp Res.

[20] Babor TF, McRee BG, Kassebaum PA, Grimaldi PL, Ahmed K, Bray J (2007). Screening, brief intervention, and referral to treatment (SBIRT) toward a public health approach to the management of substance abuse. Subst Abuse.

[21] Organization WH (2008). Task shifting: global recommendations and guidelines.

[22] Dua T, Barbui C, Clark N, Fleischmann A, Poznyak V, van Ommeren M (2011). Evidence-based guidelines for mental, neurological, and substance use disorders in low-and middle-income countries: summary of WHO recommendations. PLoS Med.

[23] Agerwala SM, McCance-Katz EF (2012). Integrating screening, brief intervention, and referral to treatment (SBIRT) into clinical practice settings: a brief review. J Psychoact Drugs.

[24] Levitt JM, Saka N, Romanelli LH, Hoagwood K (2007). Early identification of mental health problems in schools: the status of instrumentation. J Sch Psychol.

[25] American Academy of Pediatrics and others (2011). Substance use screening, brief intervention, and referral to treatment for pediatricians. Pediatrics.

[26] Källmén H, Berman AH, Jayaram-Lindström N, Hammarberg A, Elgán TH (2019). Psychometric properties of the AUDIT, AUDIT-C, CRAFFT and ASSIST-Y among Swedish adolescents. Eur Addict Res.

[27] D’Amico EJ, Parast L, Meredith LS, Ewing BA, Shadel WG, Stein BD (2016). Screening in primary care: what is the best way to identify at-risk youth for substance use?. Pediatr.

[28] Knight JR, Sherritt L, Harris SK, Gates EC, Chang G (2003). Validity of brief alcohol screening tests among adolescents: a comparison of the AUDIT, POSIT, CAGE, and CRAFFT. Alcohol Clin Exp Res.

[29] Dhalla S, Zumbo BD, Poole G (2011). A review of the psychometric properties of the CRAFFT instrument: 1999–2010. Curr Drug Abuse Rev.

[30] American Psychiatric Association Division of Research (2013). Highlights of changes from dsm-iv to dsm-5: Somatic symptom and related disorders. Focus.

[31] Mitchell SG, Kelly SM, Gryczynski J, Myers CP, O’Grady KE, Kirk AS (2014). The CRAFFT cut-points and DSM-5 criteria for alcohol and other drugs: a reevaluation and reexamination. Substance abuse.

[32] Pilowsky DJ, Wu L-T (2013). Screening instruments for substance use and brief interventions targeting adolescents in primary care: a literature review. Addict Behav.

[33] Skylstad V, Aber H, Bakken V, Dierkes J, Iversen SA, Kisaakye E (2021). Child alcohol use disorder in Eastern Uganda: screening, diagnostics, risk factors and management of children drinking alcohol in Uganda (TREAT C-AUD): a mixed-methods research protocol. BMJ Paediatrics Open.

[34] Statistics UBo. The national population and housing census 2014–Main report. Uganda Bureau of Statistics Kampala. 2016.

[35] Lwangale DW. Genealogical Reconstruction of Lubukusu, Lumasaba and Lugisu. International J Res Dev Org. 2015.

[36] Knight JR, Shrier LA, Bravender TD, Farrell M, Vander Bilt J, Shaffer HJ (1999). A new brief screen for adolescent substance abuse. Arch Pediatr Adolesc Med.

[37] Knight JR, Sherritt L, Shrier LA, Harris SK, Chang G (2002). Validity of the CRAFFT substance abuse screening test among adolescent clinic patients. Arch Pediatr Adolesc Med.

[38] Harris SK, Louis-Jacques J, Knight JR (2014). Screening and brief intervention for alcohol and other abuse. Adolesc Med State Art Rev.

[39] Sousa VD, Rojjanasrirat W (2011). Translation, adaptation and validation of instruments or scales for use in cross-cultural health care research: a clear and user-friendly guideline. J Eval Clin Pract.

[40] Yamane T. Research methods: determination of sample size. 1967.

[41] Sheehan DV, Sheehan KH, Shytle RD, Janavs J, Bannon Y, Rogers JE (2010). Reliability and validity of the mini international neuropsychiatric interview for children and adolescents (MINI-KID). J Clin Psychiatry.

[42] Abbo C, Kinyanda E, Kizza RB, Levin J, Ndyanabangi S, Stein DJ (2013). Prevalence, comorbidity and predictors of anxiety disorders in children and adolescents in rural north-eastern Uganda. Child Adolesc Psychiatry Ment Health.

[43] Nalugya-Sserunjogi J, Rukundo GZ, Ovuga E, Kiwuwa SM, Musisi S, Nakimuli-Mpungu E (2016). Prevalence and factors associated with depression symptoms among school-going adolescents in Central Uganda. Child Adolesc Psychiatry Ment Health.

[44] Kinyanda E, Kizza R, Abbo C, Ndyanabangi S, Levin J (2013). Prevalence and risk factors of depression in childhood and adolescence as seen in 4 districts of north-eastern Uganda. BMC Int Health Hum Rights.

[45] Wong HB, Lim GH (2011). Measures of diagnostic accuracy: sensitivity, specificity, PPV and NPV. Proc Singap Healthc.

[46] Dawson DA, Goldstein RB, Patricia Chou S, June Ruan W, Grant BF (2008). Age at first drink and the first incidence of adult-onset DSM-IV alcohol use disorders. Alcohol Clin Exp Res.

[47] Mitchell SG, Gryczynski J, O’Grady KE, Schwartz RP (2013). SBIRT for adolescent drug and alcohol use: current status and future directions. J Subst Abuse Treat.

[48] Kieling C, Baker-Henningham H, Belfer M, Conti G, Ertem I, Omigbodun O (2011). Child and adolescent mental health worldwide: evidence for action. Lancet.

[49] Knight JR, Sherritt L, Harris SK, Gates EC, Chang G (2003). Validity of brief alcohol screening tests among adolescents: a comparison of the AUDIT, POSIT, CAGE, and CRAFFT. Alcohol Clin Exp Res.

[50] Wartberg L, Kriston L, Diestelkamp S, Arnaud N, Thomasius R (2016). Psychometric properties of the German version of the CRAFFT. Addict Behav.

[51] Song Y, Kim H, Park SY (2019). An item response theory analysis of the Korean version of the CRAFFT scale for alcohol use among adolescents in Korea. Asian Nurs Res (Korean Soc Nurs Sci).

[52] Rial A, Kim-Harris S, Knight JR, Araujo M, Gómez P, Braña T (2019). Empirical validation of the CRAFFT abuse screening test in a Spanish sample. Adicciones.

[53] Ola B, Atilola O (2017). Validation of CRAFFT for use in youth correctional institutions in Lagos, Nigeria. J Am Acad Psychiatry Law.

[54] Pepe MS (2003). The statistical evaluation of medical tests for classification and prediction.

[55] Subramaniam M, Cheok C, Verma S, Wong J, Chong SA (2010). Validity of a brief screening instrument—CRAFFT in a multiethnic Asian population. Addict Behav.

[56] Kandemir H, Aydemir Ö, Ekinci S, Selek S, Kandemir SB, Bayazit H (2015). Validity and reliability of the Turkish version of CRAFFT substance abuse screening test among adolescents. Neuropsychiatr Dis Treat.

[57] Dancause KN, Akol HA, Gray SJ (2010). Beer is the cattle of women: Sorghum beer commercialization and dietary intake of agropastoral families in Karamoja, Uganda. Soc Sci Med.

[58] Deininger K (2003). Does cost of schooling affect enrollment by the poor? Universal primary education in Uganda. Econ Educ Rev.

[59] Van de Mortel TF (2008). Faking it: social desirability response bias in self-report research. Aust J Adv Nurs.

[60] Högberg C, Billstedt E, Björck C, Björck P-O, Ehlers S, Gustle L-H (2019). Diagnostic validity of the MINI-KID disorder classifications in specialized child and adolescent psychiatric outpatient clinics in Sweden. BMC Psychiatry.

