# Supplementary material for: Improving alcohol and substance use screening in school-age children: translation, adaptation and psychometric evaluation of the CRAFFT tool for Lumasaaba, Uganda
Source: Addict Sci Clin Pract. 2024 May 14;19:38. doi: 10.1186/s13722-024-00465-7 (PMC11095024; doi:10.1186/s13722-024-00465-7)
Supplement: Supplementary file 1 — Additional file 1: Table showing items in the original CRAFFT tool, adjustments and the final Lumasaaba version. [file 13722_2024_465_MOESM1_ESM.docx]

**Additional file 1 Table showing items in the original CRAFFT tool, adjustments and the final *Lumasaaba* version**

| **Items in original Clinician administered CRAFFT tool V 2.0** | **Adjustments made during the process** | **Reason for adjusting** | **Final Lumasaaba version of the CRAFFT tool** |
| --- | --- | --- | --- |
| Begin: "I'm going to ask you a few questions that I ask all my patients. Please be honest. I will keep your answers confidential." | After greeting and introducing yourself, start like this: "I want us to talk about alcohol, Marijuana and other drugs".  I am going to ask you a few questions that I usually ask all children and youth. Please tell me the truth. I will keep your answers confidential. *(The doctor explains what conditional confidentiality means – see next page) * "*Your responses to our questions will be kept confidential and we will not show them to your teachers or parents unless you tell us that there is someone abusing you physically or in a relationship or you have pain or you plan to hurt yourself or another person, then we might share the information with other concerned people and ask them to intervene in these issues to protect you".* Have you ever heard about something called alcohol? YES NO  Do you have friends who use alcohol or other drugs/ substances? Yes or No(If the response is 'YES' for the above questions, continue to section A below. If the response is 'NO', for both questions, stop the interview here). | The committee found it better to introduce the topic of alcohol and other substance use to the adolescent at the beginning of the session and to explain the meaning of conditional confidentiality.  Two opening questions were introduced (i) if child/adolescent has ever heard about alcohol and (ii) if they have friends who drink alcohol.  Although majority of children in our study had heard about alcohol some few had not heard about it. Therefore we found it important not to continue with interview if child has never heard about alcohol and has no friends who drink alcohol. In the opening questions we considered alcohol only because we did not find children or adolescents using marijuana or other substances without drinking alcohol. | After greeting and introducing self, Begin: "I want us to talk about alcohol, marijuana and other drugs". I’m going to ask you a few questions that I ask all children/youth. Please tell me the truth, I will keep your answers confidential*(Clinician explains meaning conditional confidentiality- see over leaf) * Conditional confidentiality:  “We will keep all your answers private, and will not show them to your teachers or parent(s) unless we believe you are in danger of being hurt.”  Have you heard about something called alcohol? (No Yes)    Do some of your friends drink alcohol? (NO, YES)  (If YES to any of the above continue with Part A questions below, if No to both stop interview here) |
| Part A  During the PAST 12 MONTHS, on how many days did you: | Part A: During the past 12 months, on how many days did you: (The doctor explains what 12 months period mean- see next page).* **The Clinician explains the meaning of 12 months period using concepts easier to understand in relation to time, for example “since last Christmas holiday”, “since last Easter holiday”, since holiday of P3, P4, or use other school events.”* | We noted that different clinicians were interpreting the 12 months period differently. Some thought it was any time in the last year. We also noted that clinicians who will use the CRAFFT tool need to be mindful on what to record in the box for frequency before proceeding to conduct the interview. | Part A  *Clinician please explain the meaning of 12 months period (see overleaf). *Clinician will explain to the child the meaning of 12 months period by using concepts easier to understand in relation to time, for example, “since last Christmas holiday”, “since last Easter holiday”, since holiday of P3, P4, or use other school events.”  **≠days** means you write the exact number of days in the box above, **put 0** if no use.  During the PAST 12 MONTHS, on how many days did you: |
| 1.Drink more than a few sips of beer, wine, or any drink containing alcohol? Put “0” if none. | 1. Drink more than a few sips (not just tasting) of beer, wine, locally made beer or any other drink containing alcohol? | Some children would not understand “sips” so we added “not just tasting”. We also added some common brands of alcoholic beverages including the locally made brands. | 1. Drink more than a few sips (not just tasting) of beer, wine, locally made beer or any other drink containing alcohol? |
| 2.Use any marijuana (weed, oil, or hash by smoking, vaping, or in food) or “synthetic marijuana” (like “K2,” “Spice”)? Put “0” if none. | 2. Use any Marijuana by chewing, or by smoking or any synthetic/ processed marijuana like cookies or sweets | We noted that marijuana use by “vaping” or synthetic marijuana “K2” were not known to the Ugandan children and adolescents. Therefore we maintained the commonly used methods (chewing and smoking) and known processed forms (Cookies, sweets). | 2. Use any Marijuana by chewing, or by smoking or any synthetic/ processed marijuana like cookies or sweets |
| 3.Use anything else to get high (like other illegal drugs, prescription or over-the-counter medications, and things that you sniff, huff, or vape)? Put “0” if none. | 3. Use any other substances to get high (other illegal drugs like Gum, nail varnish, airplane fuel, etc.)  *Get high = feeling happy, relaxed, amused, creative, altered sense of time and the way you see things. | We noted that many clinicians were not able to explain the meaning of ‘high’ in this sense and there was need to give more examples of locally available other substances that the participants mentioned. | 3. Use any other substances to get high (other illegal drugs like Gum, nail varnish, airplane fuel, etc.)  *Get high = feeling happy, relaxed, amused, creative, altered sense of time and the way you see things. |
| Did the patient answer “0” for all questions in Part A? | CLINICIAN TO READ THESE INSTRUCTIONS BEFORE CONTINUING:  If you put zero “0” in ALL of the boxes in part A above, ASK the CAR QUESTION ONLY in part B below , THEN STOP  If you did not put “0’’ in all the boxes above, ASK ALL SIX CRAFFT QUESTIONS BELOW. | We interacted with various categories of clinicians including specialists, medical doctors, psychiatric clinical officers and psychiatric nurses. Most Psychiatric clinical officers and Nurses did not understand this instruction and yet they are the ones most expected to use the tool as they are placed in the lower-level health facilities where alcohol use by children and adolescents is likely to be first identified. Therefore the instruction was detailed. | CLINICIAN TO READ THESE INSTRUCTIONS BEFORE CONTINUING:  If you put zero “0” in ALL of the boxes in part A above, ASK the CAR QUESTION ONLY in part B below , THEN STOP  If you did not put “0’’ in all the boxes above, ASK ALL SIX CRAFFT QUESTIONS BELOW. |
| If Yes  Ask CAR question only, then stop |  |  |  |
| If NO  Ask all six CRAFFT* questions below |  |  |  |
| C Have you ever ridden in a CAR driven by someone (including yourself) who was “high” or had been using alcohol or drugs? | SECTION B (Answer YES or NO)  C. Have you ever driven a CAR/bicycle/motorcycle/scooter or boda-boda while you were drunk, or driven by someone  who was drunk or high or had been using alcohol or drugs? | During the pre-testing exercise, we noted a need to repeat the instruction (answer yes or no). Since children are starting to drink at younger age and they have not yet started driving cars and even older adolescents have limited access to cars, the CAR question was adjusted to add the most available means to children and adolescents. These include bicycles, motorcycles, boda-bodas and scooters. We maintained the car for older adolescents who might have access to them. | PART B (Please answer YES or NO)  Have you ever ridden a bicycle/motorcycle/scooter or boda-boda while you were drunk, or been driven by someone who was drunk or high or had been using alcohol or drugs? |
| R  Do you ever use alcohol or drugs to RELAX, feel better about yourself, or fit in? | R. R Do you ever use alcohol or drugs to RELAX, feel better about yourself/ be able to sleep/  perform better or fit in (Not to feel shy/be accepted/ fit in group/ be same as others? | Here we added words be able to sleep, perform better, not to feel shy, be accepted in a group and be the same as others. This is according to the way Ugandan children interpreted the meaning of taking alcohol to relax. | R Do you ever use alcohol or drugs to RELAX, (feel better about yourself/ be able to sleep/perform better) or fit in group (not to feel shy/be accepted) ? |
| Do you ever use alcohol or drugs while you are by yourself, or ALONE? | A. Do you often use alcohol or other drugs ALONE (when there is no one watching you)? | Here we added “when nobody is seeing you” to help clinician understanding of the statement. | A Do you ever use alcohol or drugs while you are by yourself, or ALONE (when nobody is seeing you)? |
| F  Do you ever FORGET things you did while using alcohol or drugs? | F Do you ever FORGET (not remember) things you did when you had drunk alcohol or used drugs | to help clinician assessing child. Emphasis on forgetting the things they did when using alcohol or drugs not being asked a question when drunk and they did not remember as some put it. | F Do you ever FORGET (not remember) things you did when you had drunk alcohol or used drugs? |
| F  Do your FAMILY or FRIENDS ever tell you that you should cut down on your drinking or drug use? | F. Do your FAMILY (parents, brothers, sisters, relatives, or other people who stay in your home)  or FRIENDS ever tell you that you should reduce/stop drinking alcohol or using drugs? | For the clinician to note and be able to explain meaning of family in the Ugandan context. We also added “reduce” as our participants had been advised by a family member or friend to reduce or to stop drinking or using drugs. | F Do your FAMILY (parents, brothers, sisters, relatives, or other people who stay in your home) or FRIENDS ever tell you that you should reduce/stop drinking alcohol or using drugs? |
| T  Have you ever gotten into TROUBLE while you were using alcohol or drugs? | T. Have you ever gotten into TROUBLE while you were using alcohol or drugs? | No change was made | T Have you ever gotten into TROUBLE while you were using alcohol or drugs? |
| *Two or more YES answers suggest a serious problem and need for further assessment. | * Any use of alcohol or any drug whether or not they give any YES answers is worrisome in younger children and indicates need for further assessment. | A score of 1 Yes or higher on the six items of the Uganda version of the CRAFFT was considered an optimal cut point for screening. | * Any use of alcohol or any drug whether or not they give any YES answers is worrisome in younger children and indicates need for further assessment.  See back for further instructions ( we shall attach page 2 of the original CRAFFT tool v2.1)  NOTICE TO CLINIC STAFF/RESEARCHERS AND MEDICAL RECORDS:  The information on this page is protected by special federal confidentiality (42 CFR Part 2), which prohibit disclosure of this information unless authorized by specific written consent. A general authorization for release of medical information is NOT sufficient. |
