# Supplementary material for: Improving alcohol and substance use screening in school-age children: translation, adaptation and psychometric evaluation of the CRAFFT tool for Lumasaaba, Uganda
Source: Addict Sci Clin Pract. 2024 May 14;19:38. doi: 10.1186/s13722-024-00465-7 (PMC11095024; doi:10.1186/s13722-024-00465-7)
Supplement: Supplementary file 2 — Additional file 2 : The Lumasaaba version of the clinician administered version of the CRAFFT tool. [file 13722_2024_465_MOESM2_ESM.docx]

| THE CRAFFT INTERVIEW VERSION 2.1 FOR CHILDREN AND ADOLESCENTS AGED 6 TO 13 YEARS- UGANDA  To be orally administered by the clinician | | |
| --- | --- | --- |
| After greeting and introducing yourself, begin: I want us to talk about alcohol, marijuana, and other drugs. I’m going to ask you a few questions that I ask all children. Please tell me the truth, I will keep your answers confidential*(Clinician explains the meaning of conditional confidentiality- see overleaf) | | |
| *Have you heard about something called alcohol?* | **NO** | **YES** |
| *Do some of your friends drink alcohol?* | **NO** | **YES** |
| *If child says YES to any of the above continue with Part A questions below, if N0 to both stop interview here* | | |
| Part A  **Clinician please explain the meaning of 12 months period ( see overleaf). ≠days means you write the exact number of days in the box above, put 0 if no use* | | |
| During the PAST 12 MONTHS, on how many days did you: | | |
| 1. Drink more than a few sips (not just tasting) of beer, wine, locally made beer, or any other drink containing alcohol? | ≠days | |
| 2. Use any Marijuana by chewing, or by smoking or any synthetic/ processed marijuana like cookies or sweets? | ≠days | |
| 3. Use any other substances to get high (other illegal drugs like Gum, nail varnish, airplane fuel, etc.)  **Get high = feeling happy, relaxed, amused, creative, altered sense of time and the way you see things* | ≠days | |
| CLINICIAN TO READ THESE INSTRUCTIONS BEFORE CONTINUING:  If you put zero “0” in ALL of the boxes in part A above, ASK the C QUESTION ONLY in part B below, THEN STOP.  If you DID NOT PUT “0’’ in all the boxes above, ASK ALL SIX CRAFFT QUESTIONS BELOW. | | |
| PART B (Please answer YES or NO) | **NO** | **YES** |
| C Have you ever ridden a bicycle/motorcycle/scooter or boda-boda while you were drunk, or been driven by someone who was drunk or high or had been using alcohol or drugs? | 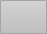 | 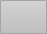 |
| R Do you ever use alcohol or drugs to RELAX, (feel better about yourself/ be able to sleep/perform better) or fit in group (not to feel shy/be accepted) ? | 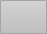 | 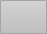 |
| A Do you ever use alcohol or drugs while you are by yourself, or ALONE (when nobody is seeing you)? | 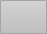 | 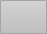 |
| F Do you ever FORGET (not remember) things you did when you had drunk alcohol or used drugs? | 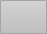 | 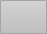 |
| F Do your FAMILY (parents, brothers, sisters, relatives, or other people who stay in your home) or FRIENDS ever tell you that you should reduce/stop drinking alcohol or using drugs? | 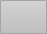 | 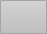 |
| T Have you ever gotten into TROUBLE while you were using alcohol or drugs? | 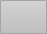 | 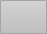 |

_________________________________________________________________________________

Any use of alcohol or any drug whether or not they give any YES answers is worrisome in younger children and indicates need for further assessment.

NOTICE TO CLINIC STAFF/RESEARCHERS AND MEDICAL RECORDS:

The information on this page is protected by special federal confidentiality (42 CFR Part 2), which prohibits disclosure of this information unless authorized by specific written consent. A general authorization for the release of medical information is NOT sufficient.

© John R. Knight, MD, Boston Children’s Hospital, 2019. Reproduced with permission from the Center for Adolescent Substance use and Addiction Research (CeASAR), Boston Children’s Hospital. For more information and versions in other languages, see [www.crafft.org](http://www.crafft.org).

* Conditional confidentiality: “We will keep all your answers private, and will not show them to your teachers or parent(s) unless we believe you are in danger of being hurt.”

*Clinician will explain to the child the meaning of 12 months period by using concepts easier to understand in relation to time, for example, “since last Christmas holiday”, “since last Easter holiday”, since holiday of P3, P4, or use other school events.”
