# Supplementary material for: Improving alcohol and substance use screening in school-age children: translation, adaptation and psychometric evaluation of the CRAFFT tool for Lumasaaba, Uganda
Source: Addict Sci Clin Pract. 2024 May 14;19:38. doi: 10.1186/s13722-024-00465-7 (PMC11095024; doi:10.1186/s13722-024-00465-7)
Supplement: Supplementary file 3 — Additional file 3 : Flow diagram showing the sampling of schools and study participants. [file 13722_2024_465_MOESM3_ESM.pdf]

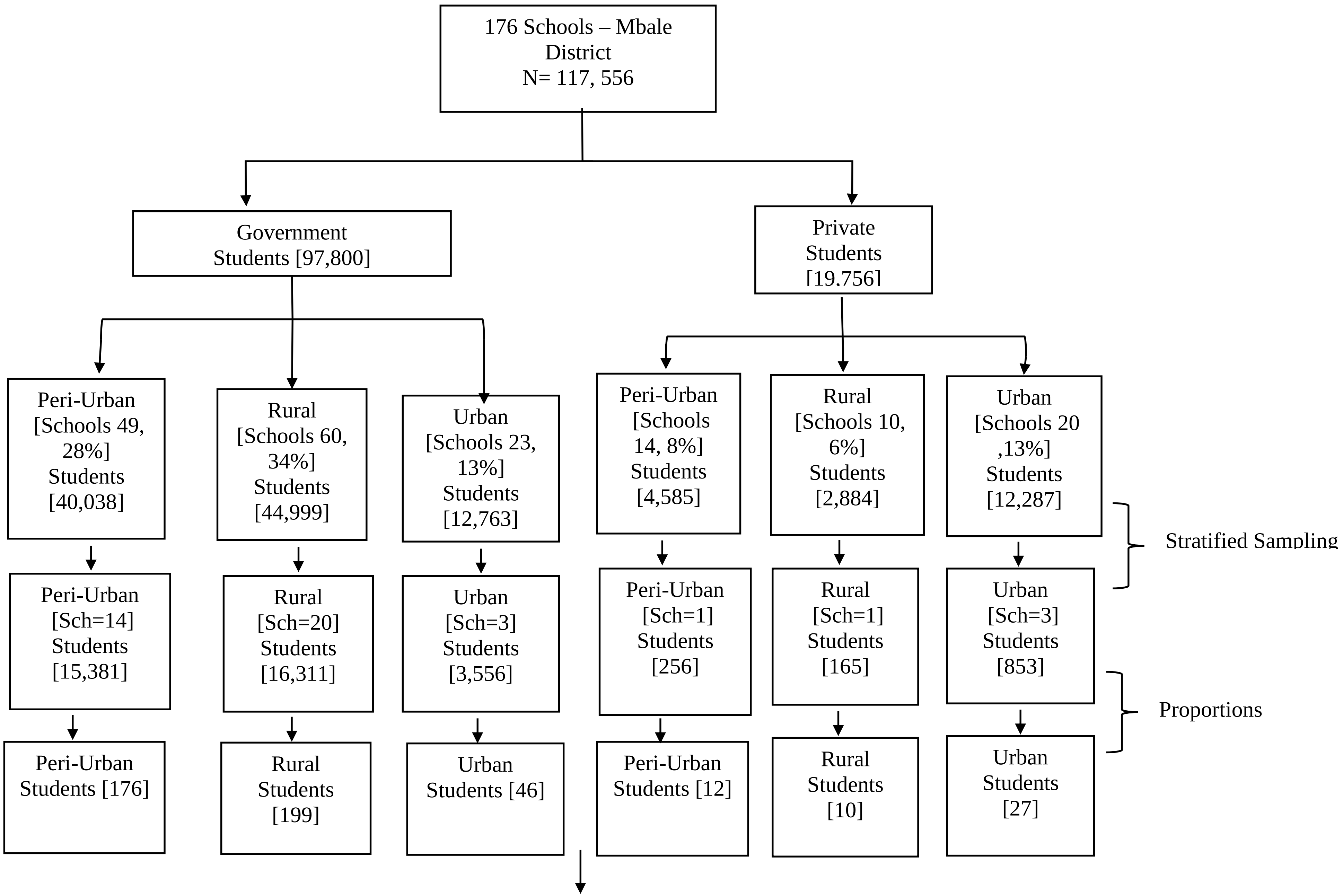

Students / pupils selection from a class for the different school by category and bands was done using simple random sampling without replacement to avoid bias for the selected/sampled school
